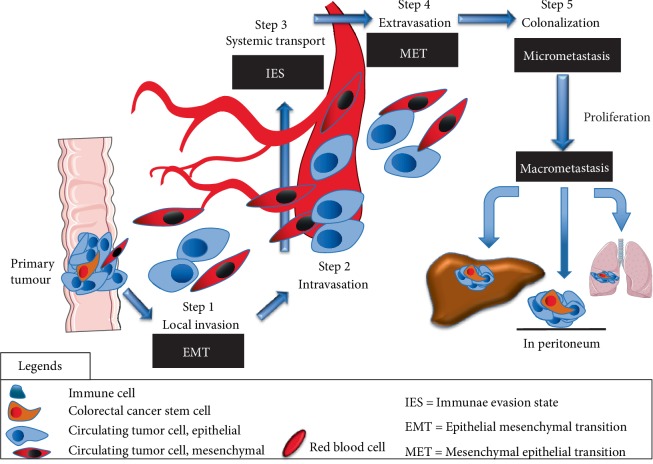# Circulating Tumour Cells, Circulating Tumour DNA and Circulating Tumour miRNA in Blood Assays in the Different Steps of Colorectal Cancer Management, a Review of the Evidence in 2019

**DOI:** 10.1155/2019/5953036

**Published:** 2019-12-04

**Authors:** Niki Christou, Jeremy Meyer, Sotirios Popeskou, Valentin David, Christian Toso, Nicolas Buchs, Emilie Liot, Joan Robert, Frederic Ris, Muriel Mathonnet

**Affiliations:** ^1^Endocrine, General and Digestive Surgery Department, CHU de Limoges, Limoges Cedex 87042, France; ^2^Laboratoire EA3842 Contrôle de l'Activation cellulaire, Progression Tumorale et Résistances thérapeutiques «CAPTuR», Faculté de médecine, 2 Rue du Docteur Marcland, 87025 Limoges, France; ^3^Department of Visceral Surgery, University Hospital of Geneva, Rue Gabrielle-Perret-Gentil 4, 1211 Genève 14, Switzerland

## Abstract

Despite many advances in the diagnosis and treatment of colorectal cancer (CRC), its incidence and mortality rates continue to make an impact worldwide and in some countries rates are mounting. Over the past decade, liquid biopsies have been the object of fundamental and clinical research with regard to the different steps of CRC patient care such as screening, diagnosis, prognosis, follow-up, and therapeutic response. They are attractive because they are considered to encompass both the cellular and molecular heterogeneity of tumours. They are easily accessible and can be applied to large-scale settings despite the cost. However, liquid biopsies face drawbacks in detection regardless of whether we are testing for circulating tumour cells (CTCs), circulating tumour DNA (ctDNA), or miRNA. This review highlights the different advantages and disadvantages of each type of blood-based biopsy and underlines which specific one may be the most useful and informative for each step of CRC patient care.

## 1. Introduction

Colorectal cancer (CRC) is a frequently diagnosed cancer in developed countries, ranking third in terms of both incidence and mortality [1].

In many countries, a bowel cancer screening program is available especially in patients with specific risks of colorectal cancer such as patients over 50 years old or hereditary colorectal cancers [2]. Common screening methods used are: (1) stool testing for blood such as guaiac fecal occult blood test or fecal immunochemical test (FIT) [3]; (2) endoscopy [4] such as rectoscopy or colonoscopy; and (3) computed tomographic (CT) colonography, which is less invasive. For many years, a reliable diagnosis has been based on biopsies from colorectal tissues. However, biopsy results alone cannot display sensitive and specific information which can provide a more complete analysis of the tumour thus allowing us to target treatment in the different phases of CRC. This is linked to the heterogeneity of tumours not only in the spatial dimension but also in the temporal one [5]. Moreover, the procedure of tissue biopsy can sometimes be invasive with risk of complications such as pain, bleeding, infections, or perforations. During screening or diagnosing colonoscopies, the overall adverse event rate has been reported to be around 2.8 per 1000 acts [6]. Even with computed tomographic (CT) colonography, a recent study including 431 Japanese centres with 147,439 CT examinations showed 0.014% of colorectal perforations [7]. In addition, tissue biopsies are also time-consuming. A lack of speed in histologic response is an ever-increasing phenomenon due in particular to the number of demands and new therapies used, for example, chemotherapy or immunotherapy.

If we look globally at prognosis, follow-up, risk of recurrence, therapeutic response, and combined clinical modalities, we see that imaging and biopsy results often lack of sensitivity and specificity while exposing the patient to specific risks.

As a result, research has been focused on developing more reliable and more accessible biomarkers. Blood, urine, cerebrospinal fluid, stool, and saliva were explored [8]. Despite, the technical difficulties and cost generated to develop those tools, progress has been made in this area. The aim of this paper is to discuss new available tools such as Circulating Tumour Cells (CTCs), Circulating Tumour DNA (ctDNA), and microRNAs (miRNAs) regarding CRC management encompassing screening, diagnosis, search for recurrences, prognosis, and prediction of therapeutic response.

## 2. Different Types of Liquid Biopsies

### 2.1. Circulating Tumour Cells (CTCs): Definition and Methods of Detection

CTCs were first described by T. Ashworth, an Australian physician, in the blood of a deceased patient [9]. They originate from both primary tumours and metastases shedding. Different biological phenotypes of CTCs exist: epithelial, mesenchymal, stem cell-like or mixed [10]. They are present in blood in very small quantities, vastly outnumbered by other cells, especially white blood cells. As a result, their detection needs a phase of isolation-enrichment and a second phase of detection.

**Figure 1:**
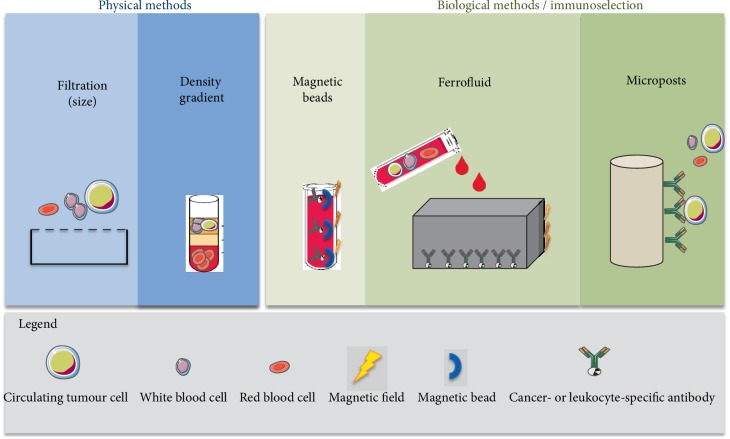


All the recent CTC identification devices combine these two steps (isolation-enrichment and detection) such as ISET [11], CellSearch System™ (Veridex, Raritan, NJ) [12], CTC-chip™ (Circulating tumour cell-chip) [13] or EPISPOT™ (EPithelial Immuno SPOT) [14]. Sometimes, different methods are used in the same device for one step: for instance, the RosetteSep™ device includes 2 methods of enrichment/isolation: by density and by immunologic separation which is a negative selection (Figure 1).

Firstly, the phase of isolation-enrichment can be performed by either physical or biological methods or by a combination of these 2 methods. Physical enrichment can be based on cell size: with a filtration system called ISET™ (Isolation by Size of Epithelial Tumour cells). It is worth noting that CTCs are larger than hematopoïetic cells. Another form of physical enrichment is possible using density gradient centrifugation (Ficoll and Percoll) (Figure 1). Immunological enrichment consists of an immunoseparation using: (1) magnetic beads, MACS (Magnetic Activated Cell Sorting) [15] with the possibility to use different kinds of antibodies; (2) ferrofluids, CellSearch [16], which separates cells bound to EpCAM-ferrofluid in a magnetic field; (3) Rosettes RARE™ (StemCell Technologies, Vancouver) (RosetteSep-Applied imaging Rare Event) [17] which combines magnetic separation with leukocyte depletion (CD 45 specific antibody); or (4) microposts, a CTC-chip which binds EpCAM-positive cells to microposts [13]. It is worth noting that typical antibodies used for enrichment are against epithelial cellular markers like EpCAM [18].

**Figure 2:**
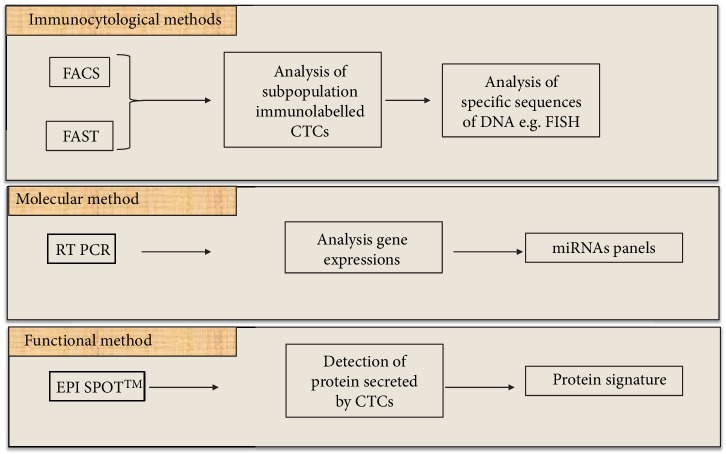


Secondly, the detection phase can be performed either at the cellular or nucleic scale (Figure 2). The cellular scale uses cytometric methods based on antigen detection, whereas the nucleic scale uses methods such RT-PCR or qRT-PCR to detect DNA or RNA alterations.

The only CTC detection system validated for clinical human use is CellSearch® [12, 19–22]. Its approval has been given by the Food Drug and Administration (FDA) and its enrichment is based on EpCAM epithelial marker detection. The FDA considers it as an aid for the monitoring of patients with metastatic CRC, breast or prostate cancer. It is key to note that detection of CTCs is most often achieved using epithelial markers such as cytokeratine [18].

The main drawback using CTCs, is that their analyses require extremely sensitive and specific methods. Firstly there is a low number of cells circulating in the blood (approximately 1 cell in 5 × 10^9^ red cells, and up to 5–10 × 10^6^ white blood cells [23]. Secondly by the use of epithelial markers, a sub-group of CTCs having presented mesenchymal–epithelial transition (MET) (Figure 3) are susceptible to being poorly detected and false-positive cells result in the detection of benign circulating epithelial cells especially during inflammatory diseases such as Crohn's disease) [24].

In most of the devices for both isolation–enrichment and detection of CTCs, epithelial markers are used because a strong relationship between epithelial positive circulating cells and prognosis of cancer has been demonstrated in many studies independent of the type of cancer [25]. Indeed, CTCs correlate to the process of metastasis. However, different types of CTCs cohabit within the circulation. Thus, tumour cells need to invade the blood circulation using Epithelial-mesenchymal transition, EMT where they have a stem cell trait [26] and then invade distant organs (mesenchymal-epithelial transition, MET). They can present either markers of EMT, like N-Cadherin, or markers of MET, such as E-cadherin [27, 28]. Furthermore, to circulate into the bloodstream, these cells have to avoid being detected by the immune system and as a result they probably face another state called the «immune-evasive state (IES)» [29].

This heterogeneity of the population of CTCs may consequently be misleading. However, we underline that detection of these cells can convey information thanks to their complete integrity, good quality, and easy to process leading proteins and nucleic acids (DNA, RNA). Thus, cytologic techniques, molecular biologic techniques and cellular cultures of live CTCs with pharmacodynamic tests can be performed.

### 2.2. Circulating Tumour DNA (ctDNA): Detection and Methods of Detection

Due to many protocols with lack of standardisation in regard to sample collections and methodologies for analyses, it is very difficult to translate this research into practical and clinical use. Briefly, it is recommended to use plasma because cfDNA serum concentration that is approximately three- to twenty-four-fold higher compared to that of plasma due to white blood cell clotting [37]. Moreover, for isolation of cfDNA it is necessary to work with specific anticoagulants such as ethylenediaminetetraacetic acid (EDTA) that has been demonstrated as the best one for analysis as it protects cfDNA from DNAses activity [38].

Because of DNAse activity, cfDNA in blood has a limited stability. It is, therefore, necessary to process the blood within 3 h after blood collection [32]. Many authors have attempted to describe homogeneous protocols such as Nikolaev et al. who have described the latest and most homogeneous [39]. A recent literature review highlights the best pre analytical conditions to study cfDNA [40]. Using a plasma sample is better than serum and EDTA or cell-free DNA™ tubes prevent lysis of blood cells. The analysis of the blood has to be done within 4 h after blood sampling. Two centrifugations are recommended and the plasma should be divided into small samples that can be stored at −80°C for up to nine months. «cfDNA extracts may sustain a maximum of three freeze–thaw cycles and storage at −20°C for up to three months for a ccfDNA (circulating cell free DNA) concentration and fragmentation analysis or up to nine months for specific sequence detection».

ctDNA can be analysed by different methods such as «targeted» (mainly using BEAMing «digital based polymerase chain reaction (dPCR)») or «nontargeted» (such as NGS: Next Generation Sequencing) methods. The dPCR array, contrary to the conventional one, is more sensitive because of the partition of DNA. NGS is a method comparing DNA sequences of normal cells and tumoural cells allowing for the discovery of new oncogenic drivers [41]. Thus, the combination of these two methods can reveal the profile of tumours and their evolution. It is an easy method using a simple blood sample collection and its analysis can assess both the tumour dynamics and the genomics modifications [42].

### 2.3. Extracellular Vesicles and MicroRNAs: Definition and Methods of Detection

Different mechanisms are known to convey communication between cells such as autocrine, paracrine or endocrine pathways, depending on the distance between the cells. Another category of cell–cell communication has been discovered through vesicles; these are defined as an extension of the cell membrane. The size varies between the type of vesicles (exosomes [30–150 nm], microvesicles [100–1000 nm], or oncosomes [1–10 *μ*m]) and they contain different factors allowing information exchanges [43]. These factors promote pathological processes, such as proliferation, metastasis [44], angiogenesis and epithelial-mesenchymal-transition [45, 46].

In CRC, exosomes have been the most studied. There is no consensus about the best method to isolate them. Ultracentrifugation, and ultrafiltration [47] are commonly used to extract them. Exosomes hold cellular components depending on the cell from which they are derived, such as proteins, DNA, mRNA, and miRNA.

MicroRNAs (miRNAs), constitute a part of noncoding RNA which represents around 80% of the complete genome. Much evidence in literature has shown that the role of non-coding RNA is fundamental concerning regulation of oncogenes and tumour suppressor genes in cancer. The small miRNAs are 18–25 nucleotides in length. Their hairpin-loop structures protect them from RNAse degradation; furthermore, their stability is enhanced not only by an embedded system with vesicles [47] or platelets [48] but also by binding with Argonaute-2. Each system of stability would be an indirect marker of cell type origin [49]. They can be extracted from different tissues or biologic fluids (circulating miRNA = cmiRNA) like feces, saliva, urine or blood [50]. This has raised the question whether miRNAs could be used as CRC biomarkers in diagnosis or prognosis. However the lack of standardization and robustness of the measuring methodology has led to contradictory results [51]. Firstly, it is difficult to extract and isolate miRNAs due to their small size and their different binding associations. Despite different ultracentrifugation methods with detergents or proteases to purify them, different variations exist highlighting the need for further studies. Secondly, various techniques are used for examination of miRNAs, such as RT-qPCR, Microarray, and Next-Generation Sequencing (NGS) with variations on sensitivity, specificity, accuracy, ease of analysis, reproducibility, and costs. RT-qPCR has the highest sensitivity and specificity. But studies have to be performed in order to improve isolation and use of miRNAs as validated biomarkers.

## 3. Early Diagnosis: Screening and Tumour Burden at Diagnosis

Up to now, clinical studies have especially emphasized ctDNA as a potential biomarker for early diagnosis and screening. Methylation of gene promoters is generally the first step in oncogenesis and according to the type of genes implicated, the origin of the tumour can be determined. As a result, many studies have focused on the research of potential signatures represented by one gene [52] or the association [53, 54] of the gene promoter's methylation profile. The best sensitivity and specificity among these marketed tests have been found for the one called Epi proColon®2.0 CE which detects methylated *Septin9* DNA [52] demonstrating 75–81% sensitivity and 96–99% specificity. By comparison, it was found that FIT had a sensitivity around 79% and a specificity near 94% [55]. Negative FIT can be preceded by the gold standard of a screening colonoscopy [56]. It is worth noting that recently, the team of Cohen et al. [57] has assessed the combined detection of circulating protein biomarkers and tumour specific mutations in circulating DNA to detect cancer in patients who already had the diagnosis of cancer (lung, ovary, stomach, colorectal, pancreas, liver, or oesophagus). The sensitivity and specificity for colon and rectum cancers using the combined detection methods was approximately 60–70%; nevertheless, this test was not conducted in healthy patients to detect colorectal cancer at a preclinical or asymptomatic stage.

The difficulty to isolate and define CTCs highlights the weakness in using them in daily clinical practice for screening and early diagnosis. Searches in the Pubmed and Cochrane databases were conducted. We searched for screening CRC, using the terms «circulating tumour cells», «screening» and «colorectal cancer», and found 1131 articles, but after analysis of each title, none corresponded to our research. We then searched for early diagnosis of CRC, using the terms «circulating tumour cells», «early diagnosis» and «colorectal cancer», and found 222 articles, but only 1 study was finally included after rigorous analysis of all the titles [58]. This article from Chen et al. [58], pointed out that extracted RNA of epithelial cell transforming sequence 2 oncogene (*ECT2*) from CTCs demonstrated a high potential to be a good diagnostic biomarker of CRC thanks to both interesting sensitivity and specificity, even in the cases were CEA concentrations accounted for less than 5 ng/mL (diagnostic threshold).

Several *in vitro* studies have highlighted specific miRNAs of CRC [59]. Recent clinical studies have confirmed the presence of these unique CRC miRNAs in comparison with healthy people [60, 61]. One recent publication by Wang et al., in 2015 [62], was able to detect a panel of 3 miRNAs in plasma, miR-409-3p, miR-7, and miR-93 as good biomarkers for CRC early detection. Other studies [63, 64], showed alternate miRNAs as potential markers for screening or early diagnosis of CRC. It is important to underline that for some studies, samples were from plasma while in others they were from serum; indeed, extracted DNA quality varies according to sample type. The most recent meta-analysis focused on blood-based miRNA analysis for diagnosis of CRC cancer was performed in 2017 by Carter et al. [65]. A literature search including articles between January 2002 and April 2016 was performed and 34 studies analysing serum or plasma miRNAs for diagnosis of CRC were included. In comparison to healthy people, the analysis showed a dysregulation of 31 miRNAs in CRC patients. Eight out of the thirty-one miRNAs were validated by more than one study: «six were up-regulated: miR-17-3p, miR-18a, miR-21, miR-29a, miR-92, and miR-106a and two were down-regulated: miR-29b and miR-145». The main issue of this meta-analysis is that a sub-group analysis according to the type of sample (either serum or plasma) was not carried out. Another meta-analysis [66] looked at 103 studies from 2008 to 2015 with 3124 CRC patients and 2579 control healthy patients. It included not only blood-based miRNAs (plasma, and/or serum) analyses but also those of tissue and feces. After meta-regression analyses, this study demonstrated that serum samples were the most accurate. Moreover, multiple miRNA assays proved to be more robust in the diagnosis of CRC in contrast to single miRNA assays [67, 68]. The accuracy was also more important in the Asian population in comparison with Caucasian people.

It appears that miRNAs are interesting prospective biomarkers but more studies are needed to confirm this in the preclinical stage, in particular within a cohort of patients who have a positive fecal immunochemical test (FIT) [69].

All in all, taking into account all the drawbacks of each biomarker, the best cancer screening method, particularly in colorectal cancer, appears at present to be CancerSEEK. It is a test developed in January 2018 by researchers in Baltimore [57] which combines the detection of ctDNA with different proteins in order to improve the signal to noise ratio.

## 4. Prognosis: Surveillance and Detection of Tumour Recurrence and Minimal Residual Disease

In colon cancers stages II and III, after radical and curative treatment, surveillance is conducted using both scanners and biological assessments (CEA biomarker) [70]. However, imaging is not sensitive enough and exposes patients to radiation while CEA does not combine high sensitivity and specificity. The best test to detect recurrences is an increase of CEA during the first year of follow-up in patients treated for CRC [71].

In rectal cancers, liquid biopsies could be of interest in order to predict the response of radiochemotherapy and disease recurrence; however, evidence in this domain is lacking.

### 4.1. Minimal Residual Disease (MRD) to Tumour Recurrences

Different studies have tried to investigate this MRD with the search of both CTCs and ctDNA after surgery. The majority of them have found a progressive decrease of ctDNA in plasma after colorectal resection for CRC in both adult women and men [72]; but the persistence of ctDNA in plasma just after resection (approximately 3 days after surgery) does not seem to correlate with further recurrences [73]. As a consequence, it is the steady decrease with time of ctDNA rates that seems to best represent the absence of residual disease. A modification with increase of ctDNA is a synonym of recurrence [74]. This has been confirmed with rectal cancers (T3/T4/N+) treated by neoadjuvant therapy [75] in samples collected before and 4–6 weeks after radiochemotherapy and surgery with sample collection 4–10 weeks postoperatively.

A clinical trial which has been initiated in our French Digestive Surgery Department (Clinical Trial: NCT02813928) aims at detecting the presence of recurrences in patients curatively treated for a CRC stages II and III within the 3 years of follow-up. This study started in July 2016 with a total of 473 patients enrolled in the follow-up of 3 years. The first results will be available at the end of 2020.

A meta-analysis [76] for CTC's, including 13 studies with patients treated for CRC with chemotherapy alone or in combination with surgery showed that high-CTC levels after treatment were correlated with disease progression confirmed by imagery.

### 4.2. Prognosis

Two recent meta-analyses have shown the impact on prognosis of both CTCs [77] and cfDNA [78] in CRC. The first, focused on CTCs [77], gathered 15 studies with 3129 patients having undergone either surgery alone or chemotherapy alone or both therapies [21, 79–92]. The presence of CTCs was linked to a poorer overall survival and progression-free disease; however there was an important heterogeneity among all the studies therefore sub-group analyses were also done. They reported that positive CTC patients had significant poor overall survival (OS) and progression-free disease (PFD) according to different criteria (time of blood collection, detection method, median follow-up and cutoff value of CTC number). Poorer OS and PFD were demonstrated for sampling collection during treatment and not at baseline, using Cellsearch detection and not RT PCR or other methods, with a median follow-up more than 24 months, and a cutoff value of CTCs superior to 1.

The second meta-analysis [78], included 9 studies of patients treated for CRC [93–101] with both qualitative and quantitative evaluation of cfDNA. Here also, there was notable heterogeneity: population size of studies, tumour stages, time of collection (8 before treatment, 1 after treatment) marker types, collection origins (plasma, *N* = 3 [93–95] or serum, *N* = 6 [96–101]), detection methods (PCR followed by sequencing [93, 96], spectrophotometry [97], quantitative PCR (q-PCR) [94, 95], mutant-enriched PCR (ME-PCR) [98], and real-time PCR (rt-PCR) [95, 99–101]. After stratification on confounding factors such as type of tumour marker searched in cfDNA, tumour stage, collection origin [serum or plasma] and methods of detection [PCR, qPCR, others] and population size, it was demonstrated that cfDNA could predict both overall survival and recurrence-free survival. Recurrences were considered as distant metastasis and confirmed by imaging.

The role of prognostic assessment by both qualitative and quantitative analysis of ctDNA was useful in CRC at a metastatic stage [102].

## 5. Therapy Response

In different cases, neoadjuvant radio chemotherapy (for middle and low rectal cancers stages II-III), or adjuvant chemotherapy (metastatic diseases or recurrences) are used.****Different combinations of drugs exist and different kinds of therapies are available: chemotherapies, targeted therapies, or immunotherapies.

Heterogeneity defines cancers and explains why for both specific tumours and stages, each person will respond differently to therapies. Analysing CTCs and ctDNA in patients with CRC allows us to choose the best therapy with respect to the tumour mutations identified: presence of RAS mutations, Anti-Epidermal Growth Factor Receptor (anti-EGFR) mutations, and BRAF mutations. Thus, the ctDNA RAS mutation profile has provided information concerning the effectiveness of the use of monoclonal antibody against EGFR such as Cetuximab [103]. Since the study of Karapetis et al. in 2008 [104], Cetuximab has been shown to improve OS and PFD in patients with metastatic CRC if they have a Wild Type RAS status. The work of Grasseli [103] has confirmed similar rates between RAS status in ctDNA (plasma) and those of tumour issue. There is also agreement towards response to anti-EGFR therapy according to RAS determination between plasma and tissue. In the same way, BRAF V600E mutation occurs in approximately 5–10% of metastatic CRCs and predicts poor prognosis [105]. As a result, therapies like BRAF and MEK inhibitors have little impact in BRAF mutated metastatic CRC. But the use of combination therapies with inhibition of EGFR and MAPK (Mitogen-activated protein kinases) pathways can improve efficacy. The study of Corcoran has proven this using anti EGFR antibody, BRAF inhibitor and MEK inhibitor with reduction in BRAF V600E mutation for 86% of patients with this triple therapy [106]. Furthermore, following the evolution of the ctDNA profile during such treatments can be used to analyse response [107] and induced genetic modifications [108]. All in all, it will help physicians to adapt these treatments over time.

A recent phase II clinical study, (PROSPECT-C trial: clinical trials.gov number NCT02994888) [109], monitored genetic variations of cfDNA in human plasma during Anti-EGFR treatment and brought to light the dynamics of resistance acquisition. This work also explained the possibility, using a mathematical model, to anticipate these variations in order to introduce personalised therapeutics.

## 6. Conclusion

Liquid biopsies looking at circulating tumour cells (CTCs), circulating tumour DNA (ctDNA), and miRNAs are of great interest in the management of CRC.

However, there is no consensus to define which may be the best combining both sensitivity and specificity for each step of CRC management due to the multiple differences in detection and analysis. It is necessary to standardise the methods of analysis for each biomarker in order to find the best sensitivity and specificity and offer the patients tailored therapies.

Moreover, we have to keep in mind that a possible multitude of markers/molecules can exist: different types of cancers use different pathways but most of the time pathways involving different molecules interlock. So our search for the “Holy Grail” of CRC management should be modified. Rather than looking for the single magical solution of CRC management and thinking that a unique method (ctDNA or CTCs or miRNAs) with a unique biomarker (in each method) for each type of cancer can exist, is wrong. The trend is rather to combine a panel of biomarkers and different methods in each step in the management of cancers in general and understanding that some identical biomarkers may be involved whatever the type of cancer. By analysing a panel of biomarkers, we can then offer optimal management for cancer diagnosis, treatment and follow-up to our patients.

## Figures and Tables

**Figure 3:**